# Longitudinal Systolic Excursion of the Mitral Annular Plane and Left Ventricular Rotational Mechanics Are Associated in Healthy Adults—Three-Dimensional Speckle-Tracking Echocardiography-Derived Insights from the MAGYAR-Healthy Study

**DOI:** 10.3390/jcm14093201

**Published:** 2025-05-05

**Authors:** Attila Nemes, Barbara Bordács, Nóra Ambrus, Csaba Lengyel

**Affiliations:** Department of Medicine, Albert Szent-Györgyi Medical School, University of Szeged, H-6725 Szeged, Hungary; bordacs.barbara.aniko@med.u-szeged.hu (B.B.); ambrusnora@gmail.com (N.A.); lecs@in1st.szote.u-szeged.hu (C.L.)

**Keywords:** left ventricular, rotation, mitral annular plane systolic excursion, three-dimensional, echocardiography

## Abstract

**Introduction**: The rotational mechanics of the left ventricle (LV) play a significant role in maintaining systemic circulation. In clinical practice, three-dimensional speckle-tracking echocardiography (3DSTE) is one of the first methods to be used that is suitable for routine, non-invasive investigations, even in healthy individuals, allowing the conduction of extensive but easily feasible tests. In routine clinical practice, mitral annular plane systolic excursion (MAPSE) is used as an easy-to-implement parameter to judge the systolic longitudinal function of the LV; its prognostic significance is also clarified. The relationship between 3DSTE-derived LV rotational mechanics and MAPSE determined by M-mode echocardiography has never been assessed. Therefore, the aim of the present study was to investigate it extensively in healthy adults. **Methods**: The present study consisted of 108 healthy adult volunteers (mean age 28.1 ± 6.3 years, 50 men). Complete two-dimensional Doppler echocardiography with MAPSE measurements and 3DSTE-derived assessment of LV rotational parameters were performed in all cases. **Results**: Both the apical and basal LV rotations and the consequential LV twist showed trends toward increase, with increasing MAPSE resulting in the LV twist being largest when the MAPSE was largest. While reduced basal LV rotation was associated with reduced MAPSE, a further increase in MAPSE with increasing basal LV rotation could not be demonstrated. With an increase in apical LV rotation, a trend toward an increase in MAPSE was seen, and was largest when the apical LV rotation was largest. No correlations could be demonstrated between MAPSE and basal LV rotation and apical LV rotation. **Conclusions**: Associations between LV longitudinal shortening, represented by MAPSE, and LV rotational mechanics could be demonstrated in healthy adults. These findings could have implications for assessing LV function in early disease states.

## 1. Introduction

The left ventricle (LV) has a central role in maintaining systemic circulation, which is aided by its complex myocardial architecture [1]. Due to the perpendicular course of the subendocardial and subepicardial LV muscle fibers, LV muscle fibers not only show a three-dimensional (3D) contraction–relaxation pattern, but the basal and apical regions of the LV also rotate in opposite directions, creating a movement similar to wringing out a towel, the so-called LV twist [2,3,4,5]. The only accepted echocardiographic tool for quantification of this movement is 3D speckle-tracking echocardiography (STE) [6,7,8,9]. During the cardiac cycle, the fibrous mitral annular (MA) plane shows a longitudinal displacement due to the contraction of the adjacent LV and left atrial (LA) myocardial tissues [10]. This sort of movement is called MA plane systolic excursion (MAPSE), the determination of which is easily performed using old-fashioned M-mode echocardiography (MME) [11,12]. To better understand the functioning of the LV, it is worth examining the relationships between the different functional characteristics, even in healthy circumstances. This may help in recognizing early cardiac dysfunction. Therefore, the present study aimed to conduct an extensive investigation between the 3DSTE-derived features of LV rotational mechanics and MAPSE as assessed by MME in healthy adults.

## 2. Subjects and Methods

**Healthy subjects**. The present study consisted of 108 healthy adult volunteers (mean age 28.1 ± 6.3 years, 50 men). All individuals were healthy due to the absence of any disorders, pathologies or other conditions in their medical history, which could affect the results. None of them presented with obesity, were professional athletes or pregnant, or smoked regularly at the time of enrollment. The physical examinations, laboratory tests, electrocardiography (ECG) and two-dimensional (2D) Doppler echocardiography performed proved to be normal in all subjects. Together with a 2D echo, 3DSTE-derived data acquisition was also performed, and the acquired data were later offline analyzed. The population of healthy individuals was grouped based on the means ± standard deviations (SDs) of certain parameters (MAPSE and basal and apical LV rotations) to reflect the physiological variability. This present retrospective study is an analysis from the ‘**M**otion **A**nalysis of the heart and **G**reat vessels b**Y** three-dimension**A**l speckle-t**R**acking echocardiography in **Healthy** subjects’ **(MAGYAR-Healthy**) **Study**. This study was partly aimed at the physiological analysis of 3DSTE-derived parameters in healthy adults, among other purposes (’Magyar’ means ’Hungarian’ in Hungarian language). The study was conducted in accordance with the Declaration of Helsinki (revised in 2013). The Institutional and Regional Biomedical Research Committee of the University of Szeged approved the study with registration number 71/2011; the latest approval was issued on 17th March 2025. All healthy individuals gave informed consent.

**Figure 1:**
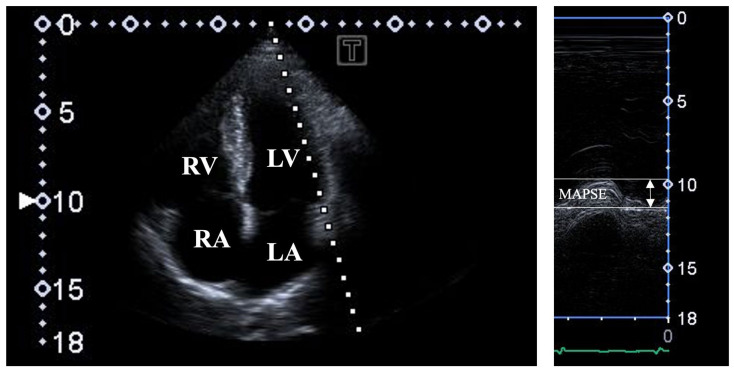
Assessment of mitral annular plane systolic excursion (MAPSE) by M-mode echocardiography in apical four-chamber long-axis view. Abbreviations: LA = left atrium; LV = left ventricle; RA = right atrium; RV = right ventricle; MAPSE = mitral annular plane systolic excursion.

**2D Doppler echocardiography**. The echocardiographic studies were performed by the same observers using a routine commercially available Toshiba Artida^TM^ echocardiography machine (Toshiba Medical Systems, Tokyo, Japan) attached to a PST-30BT (1–5 MHz) phased-array transducer. The healthy subjects lay in the left lateral decubitus position, and then the transducer was placed in the typical parasternal and apical positions for the measurements. The assessment of the LA and LV parameters followed the guidelines together with the measurement of the LV ejection fraction (EF) by Simpson’s method. Doppler echocardiography was used for valvular assessments. The transmitral early (E) and late (A) diastolic inflow velocities were measured by pulsed Doppler featuring LV diastolic function. For characterization of the longitudinal LV systolic function, MAPSE was measured by MME in the apical four-chamber (AP4CH) long-axis view as the movement of the lateral MA edge toward the LV apex [12,13] (Figure 1).

**Figure 2:**
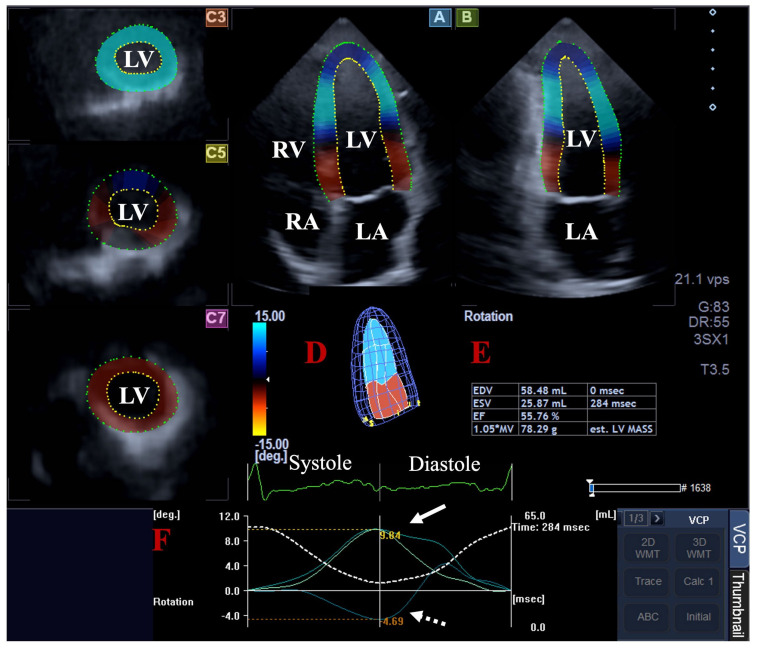
Assessment of left ventricular (LV) rotational parameters by three-dimensional (3D) speckle-tracking echocardiography. Apical four-chamber (**A**) and two-chamber (**B**) long-axis views and short-axis views at basal (**C3**), midventricular (**C5**) and apical LV levels (**C7**) are shown with virtual 3D model of LV (**D**) and calculated LV volumetric data (**E**). Time-apical (white arrow) and basal (white dashed arrow) LV rotational curves are presented together with a curve representing time–LV volume changes (dashed white line) during heart cycle (**F**). Abbreviations: LA = left atrium; LV = left ventricle; RA = right atrium; RV = right ventricle; EDV = end-diastolic volume; ESV = end-systolic volume; EF = ejection fraction.

**Three-dimensional speckle-tracking echocardiography**. The 3DSTE was carried out in 2 steps; firstly, acquisitions of the 3D echocardiographic datasets were performed using the same cardiac ultrasound tool, following a transducer change to a PST-25SX matrix phased-array transducer with 3D capability. For optimal images, 6 subvolumes within 6 cardiac cycles with a breath-hold were acquired from the apical window. As a second step, 3D Wall Motion Tracking software (version 2.7, Ultra Extend, Toshiba Medical Systems, Tokyo, Japan) was used for the analysis of the auto-merged full-volume 3D datasets. The datasets were automatically presented in 3 short-axis views at the basal, midventricular and apical LV levels and AP4CH and apical two-chamber (AP2CH) long-axis views. The observer determined the lateral and septal LV and mitral annulus edges and the endocardial surface of the LV apex in the AP4CH and AP2CH views. Then, a sequential analysis and automatic contour detection was carried out for the creation of a virtual 3D LV cast by which the LV rotational parameters were determined, including the apical and basal LV rotations, LV twist and time-to-LV twist [6,7,8,9] (Figure 2). The known limitation of the low frame rate of 3DSTE could affect the findings due to the relatively low number of frames acquired and the 3D virtual LV models created from them.

**Statistical analysis**. The variables were presented in mean ± SD or number/percentage formats, depending on whether the variable was continuous or categorical in nature. Statistical significance was present if *p* < 0.05. An independent sample *t*-test and an analysis of variance (ANOVA) test were used for data analysis using SPSS software version 22 (SPSS Inc., Chicago, IL, USA).

## 3. Results

**Clinical data**. The most important clinical data, such as the systolic and diastolic blood pressures (121.9 ± 3.1 mm Hg and 82.9 ± 2.2 mm Hg, respectively), heart rate (70.7 ± 1.7 1/s), height (168.9 ± 9.7 cm), weight (72.6 ± 14.2 kg) and body surface area (1.83 ± 0.31 m^2^) proved to be within the normal reference ranges in the healthy subject population.

**Classification of healthy individuals**. The healthy subject population was classified into subgroups according to the means ± SDs of the MAPSE and basal and apical LV rotations (Table 1). The means − SDs (11 mm, −2 degrees and 6.5 degrees, respectively) and means + SDs (17 mm, −6.2 degrees and 13.7 degrees, respectively) of these parameters served as cut-off values to create three subgroups for each parameter.

**2D Doppler echocardiography**. The routine echocardiographic parameters are presented in Table 1 and Table 2. In the MAPSE subgroups, the largest MAPSE was associated with increased LV dimensions. Reduced MAPSE was associated with increased thickness of the interventricular septum (Table 1). In the basal LV rotation subgroups, the largest basal LV rotation was associated with increased thickness of the LV posterior wall. In the apical LV rotation subgroups, the end-systolic LV dimensions were increased with reduced LV-EF when the values of the apical LV rotation were in the mean group, compared to the less-than-mean values (Table 2). All other 2D echocardiographic parameters did not differ between the subgroups (Table 1 and Table 2). None of the healthy participants had larger than grade 1 valvular regurgitation, and none of them had significant valvular stenosis.

**LV rotations in different MAPSE subgroups**. Both the apical and basal LV rotations and the consequential LV twist tendentiously increased with increasing MAPSE, resulting in the LV twist being largest when the MAPSE was largest (Table 1).

**MAPSE in different LV rotation subgroups**. While reduced basal LV rotation was associated with reduced MAPSE, a further increase in MAPSE with an increasing basal LV rotation could not be demonstrated. With an increase in apical LV rotation, a tendentious increase in MAPSE was seen, being largest when the apical LV rotation was the largest (Table 2).

**Figure 3:**
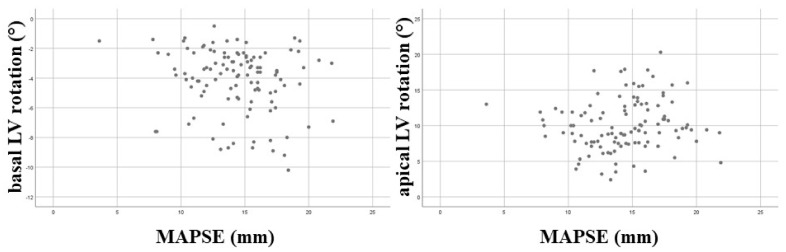
Correlations between mitral annular plane systolic excursion and basal and apical left ventricular rotations. Abbreviations: LV = left ventricular, MAPSE = mitral annular plane systolic excursion.

**Correlations**. No correlations could be demonstrated between MAPSE and basal LV rotation (r = −0.18, *p* = 0.06) or apical LV rotation (r = 0.15, *p* = 0.11) (Figure 3).

## 4. Discussion

During the cardiac cycle, the LV performs a complex contraction–relaxation pattern, which includes a longitudinal shortening in systole, narrowing circumferentially and thickening radially. At the same time, fundamentally inseparable from this movement, the LV apex rotates counterclockwise, while the LV base rotates clockwise, resulting in the LV twist. Some evidence suggests that up to 40% of the ejection is due to the LV rotational mechanics [2,3,4,5]. Another sign of systolic LV longitudinal shortening is the systolic excursion of the MA, represented by MAPSE.

Modern cardiovascular imaging techniques, including echocardiography, offer detailed, non-invasive insights into cardiac mechanics. Echocardiography still maintains its fundamental place in diagnostics due to its non-invasiveness and ease of implementation and learning [14]. The LV contractility in the longitudinal, circumferential and radial directions can be represented by objective echocardiographic features called LV longitudinal (LS), circumferential (CS) and radial strains [15]. Both 2DSTE and 3DSTE are accepted tools for the measurement of these unidimensional strains, and their normal values are available [16,17]. In addition, the well-known and validated MAPSE, with good reproducibility and a significant prognostic impact, is also used in routine clinical practice for featuring LV longitudinal movement as determined by the old-fashioned but easy-to-perform MME [11,12,18,19]. With the advent of 3DSTE, an opportunity for the non-invasive determination of LV rotational parameters and the consequential LV twist has been raised [20,21,22,23]. 3DSTE is validated and normal reference values are also available [20,21,22,23].

The complex relationship between 3DSTE-derived LV volumes, LV deformation represented by strains and LV rotational mechanics in healthy adults has already been demonstrated in the frame of the MAGYAR-Healthy Study [24,25,26]. While basal LV rotation has an inverse relationship with the global LV-CS, apical LV rotation was found to be associated with LV strains in all directions [24]. Moreover, from the LV strains, as expected, only the LV-LS showed an association with MAPSE [27]. The present study aimed to extend our knowledge in order to demonstrate the nature of the relationship between the rotational mechanics of the LV and MAPSE in healthy circumstances. It can be stated that there is a tendentious increase in the basal and apical LV rotations and LV twist with increasing MAPSE. With increasing basal LV rotation, the MAPSE increases only up to a point, beyond which a further increase could not be detected. With increasing apical LV rotation, a tendentious increase in MAPSE is present. These findings could have several implementations. First of all, the 3DSTE-derived assessment of LV rotational mechanics and MME-derived MAPSE measurement can be performed at the same time, allowing for comparative assessments. Secondly, LV volumes were not considered during our assessments, as they would have made the whole comparison very complicated, although their effects on LV rotational mechanics and MAPSE have been demonstrated [25,28]. These facts suggest the necessity for further investigations. Thirdly, the results suggest complex and regional adaptations between the LV rotational mechanics and LV longitudinal shortening represented by MAPSE, in which the basal and apical LV rotations are involved differently. This fact is in contrast with tricuspid annular plane systolic excursion, representative of right ventricular longitudinal shortening, which did not show any associations with LV rotational mechanics as demonstrated in a recent paper from the MAGYAR-Healthy Study suggesting further differences between the ventricles [29]. However, further validation studies are warranted to confirm the presented findings with other imaging techniques in a larger healthy population. Moreover, a better understanding of the LV functional characteristics in certain disorders like heart failure in different stages could be an interesting and important topic, which requires further investigation.

**Limitation section**. The most important limitations are presented here:

- One of the most important technical limitations is the low (30 ± 2 fps) frame rate of 3DSTE, which may limit the ability of 3DSTE in all measurements. Moreover, it should be taken into account that the 3DSTE-capable transducers are larger than those used for 2D Doppler echocardiography, limiting their positioning on the chest. It should also be considered that six subvolumes during six cardiac cycles are necessary for optimal image quality, potentially resulting in stitching/motion artifacts during data analysis [2,3,4,5,6].
- The 3DSTE method offers a complex volumetric and functional assessment of the LV, including strain measurements. In a recent study, the associations between 3DSTE-derived LV strains and MAPSE have been clarified; therefore, such comparisons were not performed in this study [27].
- Moreover, 3D casts can be created for all of the other chambers, and volumetric and strain analyses of both atria and the right ventricle can be performed using the same acquired 3D echocardiographic dataset. However, this study did not aim to make such evaluations.
- A more complex 3DSTE evaluation of certain valves and their annuli is an option, like ‘en-face’ measurement of the annular dimensions and the calculation of ‘sphincter-like’ functional properties. However, this study did not aim to make such assessments [30].
- Moreover, comparisons of 3DSTE with 2D Doppler echocardiography or other imaging techniques for valvular studies were not purposed, either. However, this sort of comparison would be a topic for future investigations.
- All subjects involved were considered to be healthy; however, it cannot be stated that subclinical abnormalities were excluded with 100% certainty in these subjects. Further clinical tests could have better confirmed the absence of abnormalities, although performing these tests without clinical indications could have raised ethical questions.
- Lastly, the 3DSTE-derived LV rotational parameters were not validated during the analysis due to their validated nature [20,21,22].

## 5. Conclusions

Associations between LV longitudinal shortening, represented by MAPSE, and LV rotational mechanics could be demonstrated in healthy adults.

## Figures and Tables

**Table 1:** Mitral annular plane systolic excursion and left ventricular rotational parameters in different mitral annular plane systolic excursion subgroups.

	All Subjects (*n* = 108)	MAPSE ≤ 11 mm (*n* = 14)	11 mm < MAPSE < 17 mm (*n* = 72)	17 mm ≤ MAPSE (*n* = 22)
**Two-dimensional echocardiography**				
**LA (mm)**	37.5 ± 3.7	38.2 ± 4.5	37.2 ± 3.6	37.9 ± 3.4
**LV-EDD (mm)**	47.9 ± 3.5	48.5 ± 3.8	47.4 ± 3.2	49.0 ± 4.0
**LV-EDV (mL)**	105.3 ± 23.2	110.0 ± 23.9	102.1 ± 19.5	116.0 ± 26.1 ^†^
**LV-ESD (mm)**	31.9 ± 3.1	31.6 ± 3.2	31.4 ± 2.9	33.3 ± 3.4 ^††^
**LV-ESV (mL)**	37.7 ± 9.0	37.5 ± 9.0	36.5 ± 7.5	41.6 ± 12.1 ^†††^
**IVS (mm)**	9.2 ± 1.3	9.9 ± 1.6	9.1 ± 1.2 *	9.1 ± 1.3
**LV-PW (mm)**	9.4 ± 1.5	10.0 ± 1.8	9.2 ± 1.4	9.7 ± 1.4
**LV-EF (%)**	64.5 ± 3.7	66.2 ± 3.0	64.3 ± 3.8	64.3 ± 3.3
**MAPSE (mm)**	14.4 ± 3.2	9.2 ± 1.8	14.2 ± 1.6 **	18.7 ± 1.4 ***/^††††^
**Three-dimensional speckle-tracking echocardiography**				
**apical LV rotation (degrees)**	10.1 ± 3.6	9.3 ± 2.8	10.0 ± 3.7	10.9 ± 3.5
**basal LV rotation (degrees)**	-4.1 ± 2.1	-3.8 ± 2.1	-4.0 ± 2.0	-4.9 ± 2.5
**LV twist (degrees)**	14.2 ± 4.1	13.1 ± 2.7	14.0 ± 4.0	15.8 ± 4.6 ****
**time-to-LV twist (ms)**	341.9 ± 128.5	328.3 ± 112.8	331.9 ± 132.8	384.8 ± 116

* *p* = 0.04 vs. MAPSE ≤ 11 mm; ** *p* = 0.0001 vs. MAPSE ≤ 11 mm; *** *p* = 0.0001 vs. MAPSE ≤ 11 mm; **** *p* = 0.05 vs. MAPSE ≤ 11 mm; ^†^ *p* = 0.009 vs. 11 mm < MAPSE < 17 mm; ^††^ *p* = 0.01 vs. 11 mm < MAPSE < 17 mm; ^†††^ *p* = 0.02 vs. 11 mm < MAPSE < 17 mm; ^††††^ *p* = 0.0001 vs. 11 mm < MAPSE < 17 mm. Subgroups based on mean ± standard deviation of MAPSE. Abbreviations: LV = left ventricular, EDV = end-diastolic volume, ESV = end-systolic volume, EF = ejection fraction, MAPSE = mitral annular plane systolic excursion.

**Table 2:** Mitral annular plane systolic excursion and left ventricular rotational parameters in different left ventricular rotational parameter subgroups.

	Basal LV Rotation ≤ −2 Degrees (n = 13)	−2 Degrees < Basal LV Rotation < −6.2 Degrees (n = 76)	Basal LV Rotation ≥ −6.2 Degrees (n = 19)	Apical LV Rotation ≤ 6.5 Degrees (n = 15)	6.5 Degrees < Apical LV Rotation < 13.7 Degrees (n = 75)	Apical LV Rotation ≥ 13.7 Degrees (n = 18)
**Two-dimensional echocardiography**						
**LA (mm)**	37.2 ± 4.8	37.3 ± 3.0	38.5 ± 5.0	37.2 ± 3.0	37.4 ± 3.8	37.7 ± 3.6
**LV-EDD (mm)**	47.2 ± 3.8	48.2 ± 3.6	47.6 ± 3.5	47.0 ± 3.1	48.2 ± 3.5	47.5 ± 3.4
**LV-EDV (mL)**	95.8 ± 32.5	107.3 ± 21.7	105.0 ± 19.8	100.4 ± 19.9	105.9 ± 24.2	105.0 ± 19.7
**LV-ESD (mm)**	31.0 ± 3.1	32.1 ± 3.2	31.2 ± 2.7	30.5 ± 3.0	32.2 ± 3.1 ^†^	31.5 ± 3.1
**LV-ESV (mL)**	36.5 ± 9.0	38.2 ± 9.4	37.1 ± 7.5	33.2 ± 7.5	38.6 ± 9.6 ^††^	37.6 ± 6.6
**IVS (mm)**	9.0 ± 1.5	9.3 ± 1.2	9.4 ± 1.4	8.9 ± 1.5	9.3 ± 1.3	9.2 ± 1.3
**LV-PW (mm)**	9.0 ± 1.1	9.3 ± 1.5	10.1 ± 1.7 ^#^	9.4 ± 1.6	9.4 ± 1.6	9.6 ± 1.1
**LV-EF (%)**	65.3 ± 2.8	64.4 ± 3.9	64.6 ± 3.1	66.9 ± 4.0	64.0 ± 3.6 ^†††^	64.6 ± 2.8
**MAPSE (mm)**	12.3 ± 3.9	14.6 ± 2.7 *	14.8 ± 3.7	13.8 ± 2.1	14.2 ± 3.4	15.6 ± 1.9 ^†††††††^
**Three-dimensional speckle-tracking echocardiography**						
**apical LV rotation (°)**	10.7 ± 3.6	10.1 ± 3.3	9.4 ± 4.5	4.8 ± 1.2	9.8 ± 1.9 ^††††^	16.0 ± 1.7 ^††††††††^/^‡^
**basal LV rotation (°)**	-1.5 ± 0.3	-3.6 ± 1.0 **	-8.0 ± 0.9 ****/^##^	-5.0 ± 2.2	-3.9 ± 2.0 ^†††††^	-4.3 ± 2.4
**LV twist (°)**	12.2 ± 3.7	13.8 ± 3.4	17.4 ± 4.9 *****/^###^	9.9 ± 2.9	13.7 ± 2.6 ^††††††^	20.3 ± 3.3 ^†††††††††^/^‡‡^
**LV twist time (ms)**	270.1 ± 97.2	352.1 ± 137.2 ***	350.2 ± 91.7 ******	297.2 ± 173.9	346.6 ± 122.1	356.1 ± 111.3

* *p* = 0.01 vs. basal LV rotation ≤ −2 degrees; ** *p* = 0.0001 vs. basal LV rotation ≤ −2 degrees; *** *p* = 0.04 vs. basal LV rotation ≤ −2 degrees; **** *p* = 0.0001 vs. basal LV rotation ≤ −2 degrees; ***** *p* = 0.0001 vs. basal LV rotation ≤ −2 degrees; ****** *p* = 0.03 vs. basal LV rotation ≤ −2 degrees; ^#^ *p* = 0.05 vs. −2 degrees < basal LV rotation < −6.2 degrees; ^##^ *p* = 0.0001 vs. −2 degrees < basal LV rotation < −6.2 degrees; ^###^ *p* = 0.0003 vs. −2 degrees < basal LV rotation < −6.2 degrees; ^†^ *p* = 0.05 vs. apical LV rotation ≤ 6.5 degrees; ^††^ *p* = 0.04 vs. apical LV rotation ≤ 6.5 degrees; ^†††^ *p* = 0.007 vs. apical LV rotation ≤ 6.5 degrees; ^††††^ *p* = 0.0001 vs. apical LV rotation ≤ 6.5 degrees; ^†††††^ *p* = 0.05 vs. apical LV rotation ≤ 6.5 degrees; ^††††††^ *p* = 0.0001 vs. apical LV rotation ≤ 6.5 degrees; ^†††††††^ *p* = 0.05 vs. apical LV rotation ≤ 6.5 degrees; ^††††††††^ *p* = 0.0001 vs. apical LV rotation ≤ 6.5 degrees; ^†††††††††^ *p* = 0.0001 vs. apical LV rotation ≤ 6.5 degrees; ^‡^ *p* = 0.0001 vs. 6.5 degrees < apical LV rotation < 13.7 degrees; ^‡‡^ *p* = 0.0001 vs. 6.5 degrees < apical LV rotation < 13.7 degrees. Subgroups based on means ± standard deviations of basal and apical LV rotations. Abbreviations: LV = left ventricular, EDV = end-diastolic volume, ESV = end-systolic volume, EF = ejection fraction, MAPSE = mitral annular plane systolic excursion.

## Data Availability

All of the data are available. This author takes responsibility for all aspects of the reliability and freedom from bias of the data presented and their discussed interpretations.
